# Efficient Haplotype Block Partitioning and Tag SNP Selection Algorithms under Various Constraints

**DOI:** 10.1155/2013/984014

**Published:** 2013-11-11

**Authors:** Wen-Pei Chen, Che-Lun Hung, Yaw-Ling Lin

**Affiliations:** ^1^Department of Applied Chemistry, Providence University, Taichung 433, Taiwan; ^2^Department of Computer Science and Communication Engineering, Providence University, Taichung 433, Taiwan; ^3^Department of Computer Science and Information Engineering, Providence University, Taichung 433, Taiwan

## Abstract

Patterns of linkage disequilibrium plays a central role in genome-wide association studies aimed at identifying genetic variation responsible for common human diseases. These patterns in human chromosomes show a block-like structure, and regions of high linkage disequilibrium are called haplotype blocks. A small subset of SNPs, called tag SNPs, is sufficient to capture the haplotype patterns in each haplotype block. Previously developed algorithms completely partition a haplotype sample into blocks while attempting to minimize the number of tag SNPs. However, when resource limitations prevent genotyping all the tag SNPs, it is desirable to restrict their number. We propose two dynamic programming algorithms, incorporating many diversity evaluation functions, for haplotype block partitioning using a limited number of tag SNPs. We use the proposed algorithms to partition the chromosome 21 haplotype data. When the sample is fully partitioned into blocks by our algorithms, the 2,266 blocks and 3,260 tag SNPs are fewer than those identified by previous studies. We also demonstrate that our algorithms find the optimal solution by exploiting the nonmonotonic property of a common haplotype-evaluation function.

## 1. Introduction

Single-nucleotide polymorphisms (SNPs) play important roles in disease association studies owing to their high abundance in the human genome, low mutation rate, and amenability to high-throughput genotyping. A small subset of SNPs directly influences the quality or quantity of the gene product and increases the risks of certain diseases or of severe side effects of drugs. Alleles of SNPs that are close together tend to be inherited together. A haplotype refers to a set of SNPs found to be statistically associated on a single chromosome. Haplotypes defined by common SNPs have important uses in identifying disease association and human traits [1–5].

Genome-wide association studies based on linkage disequilibrium (LD) offer a promising approach to detect genetic variation responsible for common human diseases. The patterns of linkage disequilibrium (LD) observed in human chromosome show a block-like structure [1, 6, 7], such that the entire chromosome can be partitioned into high-LD regions interspersed with low-LD regions. The high-LD regions are called haplotype blocks and the low-LD regions are referred to as recombination hotspots. A few common haplotypes account for most of the variation from person to person in haplotype blocks. Furthermore, each haplotype block, comprising large regions of low diversity, can be characterized with a small number of SNPs, which are referred to as tag SNPs [8]. Identification of tag SNPs is aimed at tagging candidate genes which can capture the most information in the haplotype blocks. The new technologies allow to genotype rarer variants than before [9]; therefore, there are more and more genotyping data needed to be analyzed, and the structure of haplotype blocks will be more complicated. Despite great progress in current genotyping developments which allow intensive genotyping at cheap prices, the concept of tag SNP selection is more and more significant due to exploded genotyping data. Most tag SNP selection strategies are based on haplotype blocks and have the aim of identifying a minimal subset of SNPs able to tag the most common haplotypes [7, 10].

Several methods have been used to identify haplotype-block structures, including LD-based [6, 11], recombination-based [12, 13], information-complexity-based [14–16], and diversity-based [7, 17, 18] methods. The result of block partitioning and the meaning of a haplotype block may differ according to the assessment criteria. The diversity-based test methods can be classified into two categories: those that divide strings of SNPs into blocks on the basis of the decay of LD across block boundaries and those that delineate blocks on the basis of some haplotype-diversity measure within the blocks. Patil et al. [7] defined a haplotype block as a region in which a fraction of a percent or more of all the observed haplotypes are represented at least *n* times or at a given threshold in the sample. They applied the optimization criteria outlined by Zhang et al. [10, 19] and described a general algorithm that defines block boundaries in a way that minimizes the number of SNPs that are required to identify all the haplotypes in a region. They identified a total of 4,563 tag SNPs and 4,135 blocks defining the haplotype structure of human chromosome 21 [7]. In each block they required that at least 80% of the haplotypes must be represented more than once in the block.

In this paper, we propose two dynamic programming algorithms, incorporating several haplotype diversity evaluation functions, for haplotype block partitioning with constraints on diversity and the number of tag SNPs. Part of data related to this paper have been open to public domain http://www.cs.pu.edu.tw/~yawlin/ (web-site page of a coauthor), these data are also published at local conference [20].

## 2. Diversity Functions

Several operational definitions have been used to identify haplotype-block structures [6, 7, 11–18] and result in different results of block partitioning and meanings of a haplotype block, depending on the objective function. Haplotype blocks are genome regions with high LD, so that distinct haplotype patterns within the block are few and the diversity of the block is low. In terms of diversity functions, the block selection problem can be viewed as finding a segmentation of a given haplotype matrix such that the diversities of chosen blocks satisfy a certain value constraint. We use the following definitions.

**Definition 1 (haplotype block diversity)**

Given an interval [*i*, *j*] of a haplotype matrix **A** and a diversity function, *δ* : [*i*, *j*] → *δ*(*i*, *j*) ∈ **R** is an evaluation function measuring the diversity of the submatrix **A**(*i*; *j*).

Given an *m* × *n* haplotype matrix *A*, a block *B*(*i*, *j*) of matrix *A* is viewed as *m* haplotype strings; they are partitioned into groups by merging identical haplotype strings into the same group. The probability *p*_*i*_ of each haplotype pattern *b*_*i*_ is defined accordingly, such that ∑*p*_*i*_ = 1. Li [21] proposes a diversity function defined by

(1) $$\begin{array}{c} \delta_{D}\left(B\right)=1-\sum_{b_{i}\in B}p_{i}^{2}. \end{array}$$

Note that *δ*_*D*_(*B*) is the probability that two haplotype strings chosen at random from *B* are different from each other. Other measurements of diversity can be obtained by choosing different diversity functions; for example, to measure information complexity one can choose the information entropy (negative-log) function [14–16] as follows:

(2) $$\begin{array}{c} \delta_{E}\left(B\right)=-\sum_{b_{i}\in B}p_{i}\log p_{i}. \end{array}$$

Patil et al. [7] and Zhang et al. [10, 18] define a haplotype block as a region where at least 80% of observed haplotypes within a block must be common haplotypes. Using the same definition of common haplotype, the coverage of common haplotypes of the block can be formulated as a form of diversity as follows:

(3) $$\begin{array}{c} \delta_{C}\left(B\right)=1-\frac{\sum_{s_{i}\in C}p_{i}}{\sum_{s_{i}\in U}p_{i}}=\frac{\sum_{s_{i}\in M}1/m}{\sum_{s_{i}\in U}p_{i}}, \end{array}$$

where *U* denotes unambiguous haplotypes, *C* denotes common haplotypes, and *m* denotes singleton haplotypes. In other words, Patil's method requires that *δ*_*C*_(*B*) ≤ 20%.

Some methods [6, 22, 23] presented a definition of a haplotype block based on the LD measure *D*′; however, there is no consensus definition as yet. Zhang and Jin [22] defined a haplotype block as a region in which no pairwise |*D*′| values are lower than a threshold *α*. Let *S* denote a haplotype interval [*i*, *j*]. We define the diversity as the complement of minimal |*D*′| of *B*. By this definition, *B* is a haplotype block if its diversity is lower than 1 − *α*. Consider

(4) $$\begin{array}{c} \delta_{L1}\left(B\right)=1-\min\left\{\left(\left|D_{i^{\prime }j^{\prime }}^{\prime }\right|\right)\;|\;i<i^{\prime }<j^{\prime }<j\right\}. \end{array}$$

Zhang et al. [22] also propose a definition for haplotype block; they require a proportion at least *α* of SNP pairs having strong LD (pairwise |*D*′| greater than a threshold) in each block. We use this definition to redefine the function as the proportion of SNP pairs without strong LD. *N*(*i*, *j*) denotes the number of SNP pairs without strong LD in the interval [*i*, *j*]. The diversity function is as follows:

(5) $$\begin{array}{c} \delta_{L2}\left(B\right)=\frac{N_{\left(i,j\right)}}{\left(\begin{array}{c} \left(j-i\right)+1\\ 2 \end{array}\right)}=\frac{N_{\left(i,j\right)}}{\left(1/2\right)\left[{\left(j-i\right)}^{2}+j-i\right]}. \end{array}$$

Diversity measurement usually reflects recombination events that occurred during evolution. Generally, haplotype blocks with low diversity indicate conserved regions of the genome.

**Definition 2 (monotonic diversity)**

A diversity function *δ* is said to be monotonic if for any haplotype block (interval) *I* = [*i*, *j*] of haplotype matrix **A**, it follows that *δ*(*i*′, *j*′) ≤ *δ*(*i*, *j*) whenever [*i*′, *j*′]⊂[*i*, *j*]; that is, the diversity of any subinterval of *I* is no larger than the diversity of *I*.

**Figure 1:**
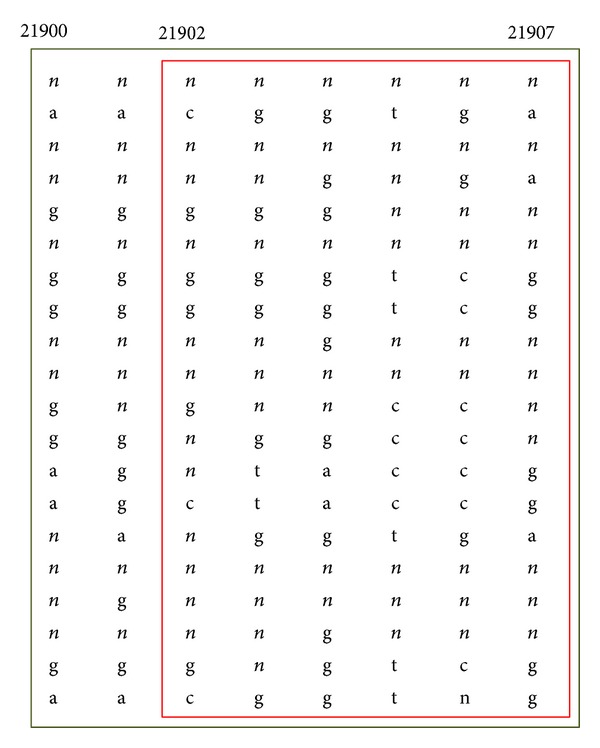
The evaluation function of common haplotypes does not satisfy the monotonic property when the haplotype sample has missing data.

The diversity functions (1) and (2) are monotonic. However, the evaluation function for common haplotypes proposed by Patil et al. [7] does not satisfy the monotonic property when the haplotype sample has missing data. For example, Figure 1 shows a small portion of human chromosome 21 haplotype sample provided in [7], where *n* denotes missing data. In the sample, the common-haplotype coverage of interval [21900, 21907] is 9/10, which is greater than 80%. Therefore, according to the definition, it is a feasible haplotype block. In contrast, the common haplotype coverage of interval [21902, 21907] is 3/7, which is less than 80%, so that it is not a feasible haplotype block. Both interval [21900, 21907] and interval [21902, 21907] terminate at the same SNP locus, and the interval [21900, 21907], which has more SNPs, is a feasible haplotype block, whereas interval [21902, 21907] is not. Tag SNPs can capture most of the haplotype diversity in blocks and thereby could potentially capture most of the information about association between a trait and the SNP marker loci. The diversity and features of each haplotype block can be described easily and economically with tag SNPs. For these reasons, we want to define the haplotype structure using as few tag SNPs as possible. In previous studies, Patil et al. [7] defined a haplotype block as a region in which a fraction of percent or more of all the observed haplotypes are represented at least *n* times or at a given threshold in the sample. They applied the optimization criteria outlined by Zhang et al. [10, 18] and described a general algorithm that defined block boundaries in a way that minimizes the number of tag SNPs that are required to distinguish uniquely a certain percentage of all the haplotypes in a region. The greedy algorithm [7] identified a total of 4,563 tag SNPs and a total of 4,135 blocks to define the haplotype structure of human chromosome 21. In each block, they required that at least 80% of haplotypes are represented more than once in the block. In addition, Zhang et al. [10] used a dynamic programming approach to reduce the numbers of blocks and tag SNPs to 2,575 and 3,582, respectively.

Both of the algorithms [7, 10] fully partition the haplotype sample into blocks with the objective of minimizing the tag SNPs. However, when the resources are limited, investigators and biologists may be unable to genotype all the tag SNPs and instead must restrict the number of tag SNPs to be identified by the algorithms. In this paper, we propose two dynamic programming algorithms for the haplotype-block partitioning problem.

**Problem 3 (longest-*k*-blocks)**

Given a haplotype matrix **A** and a diversity upper limit **D**, we wish to find *k* disjoint blocks whose diversity is less then D such that the total length is maximized. That is, output the set *S* = {*B*_1_, *B*_2_,…, *B*_*k*_}, with *δ*(*B*) ≤ *D* for each *B* ∈ *S*, such that |*B*_1_| + |*B*_2_| + ⋯+|*B*_*k*_| is maximized. Here |*B*_*i*_| denote the length of block *B*_*i*_.

**Figure 2:**
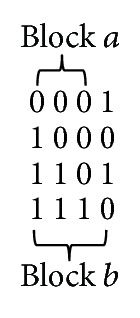
Illustration of the idea of good partner.

Assuming that the given diversity function is monotonic and the given haplotype matrix is preprocessed for finding the indices of the farthest site, called *good partner* site, indices from current site, the longest-*k*-block problem can be solved in *O*(*n*) space and *O*(*kn*) time. The *good partner* of locus *i* refers to the left farthest locus from *i*, *Li* such that [*Li*, *i*] is a haplotype block whose diversity is less then the upper limit constraint. The idea of left good partner is shown in Figure 2.

**Problem 4 (longest-blocks-*t*-tags)**

Given a haplotype matrix **A** and a diversity upper limit **D**, we wish to find a list of disjoint blocks whose total tag SNP number is less than *t* such that the total length is maximized. That is, output the set *S* = {*B*_1_, *B*_2_,…, *B*_|*S*|_} such that (for all *B*_*i*_ ∈ *S*) (*δ*(*B*_*i*_) ≤ *D*) and ∑tag(*B*_*i*_) ≤ *t*; tag(*B*_*i*_) denote the number of tag SNPs required for block *B*_*i*_, so that |*B*_1_ | +|*B*_2_ | +⋯+|*B*_|*S*|_| is maximized. Here |*B*_*i*_| denote the length of block *B*_*i*_.

Assuming that all of the feasible blocks and tag SNPs required for each block have been preprocessed, the longest-blocks-*t*-tags problem can be solved in *O*(*tL*) time, where *L* denotes the total number of feasible blocks. For the same sample used, based on the same criteria adopted by [23], our algorithm identifies a total of 2,266 blocks, which can be tagged by 3,260 tag SNPs. The number of blocks and tag SNPs we identified are 45.2% and 28.6% fewer than those identified by [7]. These results are also better than those by Zhang's method with respect to the number of tag SNPs used and the total block numbers.

The definition of the haplotype-block diversity evaluation function (*δ*) we use in this paper is equal to the ratio of singleton haplotypes to unambiguous haplotypes in the blocks. It is also equal to 1 minus the ratio of common haplotypes to unambiguous haplotypes; in other words, 80% of common-haplotype coverage is equal to 20% (or 0.2) of haplotype diversity by the definition presented in [7]. That is, we require the diversity of each block to be ≤0.2. Here we propose two linear-space algorithms for these two problems.

## 3. Method

We propose two dynamic programming algorithms to partition haplotype blocks with constraints on diversity and number of tag SNPs. The proposed algorithms are able to find the longest segmentation *S* into blocks such that the diversity of each block is less than an upper limit *D* and the total number of tag SNPs required for these blocks does not exceed a specified number *t*, and they are time-efficient and linear-space algorithms for solving Problems 3 and 4. In the first algorithm, the longest segmentation consisting of *k* feasible blocks can be found in *O*(*kn*) time and linear space after the preprocessing of the leftmost site *L*[*i*] (good partner site) and the rightmost site *R*[*i*] for each SNP marker *i*. After partitioning blocks, we select tag SNPs in each block. Using this method, we can partition a haplotype into a minimum number of blocks with a modest number of tag SNPs. In the second algorithm, the longest segmentation covered by *t* tag SNPs can be found in *O*(*tL*) time after the preprocessing of left good partners *L*[*i*] for each marker *i* and tag SNPs required for each feasible block. Using this method, we can partition a haplotype into a minimum number of blocks with a minimum number of tag SNPs.

### 3.1. Tag SNP Selection Algorithm

Our algorithms begin with the preprocessing of the farthest site (good partner) for each SNP marker. According to the haplotype block definition defined by [7], at least 80% of unambiguous haplotypes must be represented more than once. Using the same criteria as in [7], for each block we want to minimize the number of SNPs that distinguish uniquely at least 80% of the unambiguous haplotypes in the block. Those SNPs can be thought of as a signature of the haplotype-block partition.

**Figure 3:**
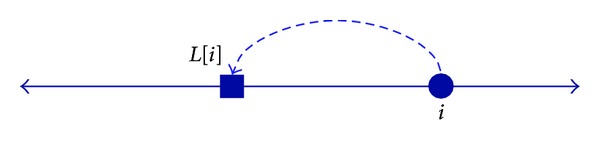
An example of a long block requiring only a few tag SNPs.

In general, the number of tag SNPs required increases as the length of the haplotype block increases. But an exception is shown in Figure 3. The block consisting of 3 SNPs needs 3 tag SNPs to distinguish each haplotype uniquely, but the block *b* consisting of 4 SNPs needs only 2 tag SNPs (column 2 and column 4).

The problem of finding the minimum number of tag SNPs within a block that uniquely distinguishes all the haplotypes is known as the MINIMUM TEST SET problem and has been proven to be NP complete [24]. Thus, there is no polynomial-time algorithm that guarantees to find the optimal solution for any input, though approximate, greedy algorithms have been proposed [25–27]. In order to find the optimal solution, we adopt a brute-force method to find tag SNPs within a block. Our strategy for selecting the tag SNPs in haplotype blocks is as follows. First, the common haplotypes are grouped into *k* distinct patterns by merging the compatible haplotypes in each block. After the missing data are assigned in each group, we determine the smallest number of tag SNPs required based on the smallest number of haplotype groups needing to be distinguished such that haplotypes in these groups contain at least 80% of the unambiguous haplotypes in the block. Finally, we select a locus set consisting of the minimum number of SNPs in the haplotypes such that at least 80% of the unambiguous haplotypes can be uniquely distinguished. An exhaustive search can be used very efficiently, given that the number of tag SNPs needed for each block is usually modest. The exhaustive-search algorithm shown in Algorithm 1 enumerates the *t*-combinations in lexicographic order to generate the next candidate tag SNP set until each pattern can be uniquely distinguished.

### 3.2. A Linear-Space Algorithm for Haplotype Block Partitioning

**Figure 4:**
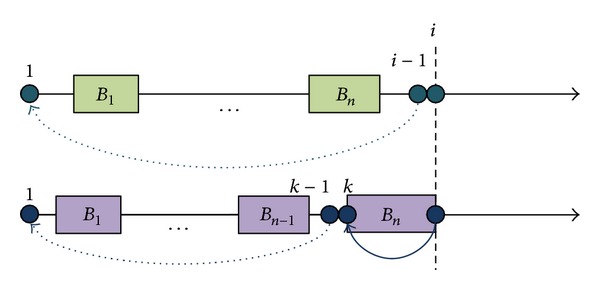
The idea behind the Zhang et al.'s dynamic programming algorithm.

Patil et al. [7] studied the global haplotype structure on chromosome 21 and identified 20 haplotypes for 24,047 SNPs (MAF ≥ 0.1) spanning over about 32.4 Mbps. By the sample, they applied a greedy algorithm to partition the haplotype into blocks of limited haplotype diversity. Using the same criteria as in Patil et al., Zhang et al. [18, 23, 28] provided a dynamic programming algorithm to partition the same sample totally into 2.575 blocks and identify a total of 3,582 tag SNPs that are 37.7% and 21.5% smaller, respectively, then those identified by Patil et al. The space complexity for Zhang et al.'s algorithm is *O*(*t* · *n*) and the time complexity is *O*(*N* · *t* · *n*), where *t* is the total number of tag SNPs, *n* is the total length of haplotype sample, and *N* is the number of SNPs contained in the largest block. The idea behind the Zhang et al.'s algorithm is illustrated in Figure 4. The maximized segmentation *S* consisting of *n* disjoint blocks between sites 1 and *i* with the constraint of using at most *t* tag SNPs will have two cases, either the site *i* is included in the last block of *S* or not. If site *i* is not included in the last block of *S*, it will find *S* between sites 1 and *i* − 1; otherwise there will exist a site *k*, 1 ≤ *k* ≤ *i*, such that [*k*, *i*] is the last block of *S*. In the latter case, the tag SNPs required for block [*k*, *i*] is tag(*k*, *i*), so it can find other blocks which are covered by other *t* − tag(*k*, *i*) tag SNPs between site 1 and site *k* − 1.

Assuming a monotonic diversity function, the recurrence relation is

(6) f(k,1,j)=max⁡{f(k,1,j−1),f(k−1,1,L[j]−1)+j−L[j]+1}.

The idea behind the recurrence relation is that either the *k*th block of the maximal segment *S* in [1, *j*] does not include site *j* or the block [*L*[*j*], *j*] must be the last block of *S*. Note that *f*(*k*, 1, *j*) can be determined in *O*(1) time if all of the *f*(*k* − 1, 1, …) and *f*(*k*, 1, 1 ⋯ (*j* − 1)) have been calculated. It follows that *f*((*k*, 1, …))'s can be calculated from the (*k* − 1, 1,…) in *O*(*n*) time. Thus a computation proceeding from the *f*(1, 1,…), *f*(2, 1, …), …, to the *f*(*k*, 1,…) takes *O*(*nk*) time.  Lemma 5 presents the dynamic programming theory for the general case.

**Lemma 5**

Given a submatrix **A**′(*i*, *j*) of an *m* × *n* haplotype matrix **A** and a diversity upper limit **D**, for all constrained intervals [*i*, j*], *i* ≤ j* ≤ *j*, find a segmentation consisting of k feasible blocks such that the total length can be maximized in *O*(|*j* − *i* | *k*) time after the preprocessed leftmost markers (tag SNP selection), *L*[*i*]'s are prepared.

Finding a segmentation that consists of *k* feasible blocks and maximum total length can be completed using dynamic programming based on the recurrence relation. However, it is difficult to retrieve the *k* intervals in linear space. To solve this problem, we can use a concept similar to that of [29]. We find a cut point x* to divide *n* SNP sites into two parts, *n*_1_ and *n*_2_, and then there are ⌊*k*/2⌋ blocks in *n*_1_ and ⌈*k*/2⌉ blocks in *n*_2_ and *n*_2_ = *n* − *n*_1_. We now have the following recursion relation. While *k* = 1, the boundaries of the block can be found by scanning the leftmost marker array and appending the longest feasible block in [*i*, *j*] to a global data structure. The algorithm is shown in Algorithm 2.

**Theorem 6 (longest-*k*-blocks)**

Given a haplotype matrix A and a diversity upper limit D, the longest k-block and their boundaries can be computed in *O*(*nk*) time and *O*(*n*) space after the preprocessed left- and rightmost markers, *L*[*i*] and *R*[*i*] are prepared.

**Proof**

We propose an *O*(*nk*) time algorithm, Lis(*k*, *i*, *j*), shown in Algorithm 1. Note that *O*(*mn*) time suffices for preprocessing to find the rightmost markers *R*[*i*] and leftmost markers *L*[*i*] for each marker site *i* as shown in [30].

In this algorithm, we use six global data structures involving arrays *L*, *R*, *A*, *B*,C, and *Y-list*. *L* and *R* are used to store the good partner points *L*[*i*] and *R*[*i*] that have been calculated in preprocessing. *Y-list* is used to store the boundaries of *k* blocks. Arrays *A* and *B* are used to store the results of the *f*(⌊*k*/2⌋, *i*, *x*) and *f*(⌈*k*/2⌉, *x* + 1, *j*). During the computation of the *f*(⌊*k*/2⌋, *i*, *x*) and the *f*(⌈*k*/2⌉, *x* + 1, *j*), we use array *C*, replacing a *k* × *n* table to store temporary results that will be used to calculate further results. The size of each of arrays *R*, *L*, *A*, *B*, and *C* is *n*. The size of *Y-list* is *k*, *k* × *n* in the general case, so that the space used by the algorithm is *O*(*n*).

The time complexity of the algorithm is *O*(*nk*) as shown in the following by induction. Let *T*(*n*, *k*) denote the time needed for Lis(*k*, 1, *n*). Assume that *T*(*n*′; *k*′) ≤ *c*_2_ *n*′*k*′ for all *n*′ < *n*,  *k*′ < *k*. According to the algorithm, we have

(7) T(n,k)=c1nk+T(n1,⌊k2⌋) +T(n−n1,⌈k2⌉).

By induction,

(8) T(n,k)≤c1nk+c2n1⌊k2⌋+c2(n−n1)⌈k2⌉≤(c1k+c2⌈k2⌉)n+c2n1⌊k2⌋−c2n1⌈k2⌉≤(c1k+c2⌈k2⌉)n, where  k≥2,  ⌈k2⌉≤23k≤c2nk.

Letting *c*_2_ = 3*c*_1_, the above inequality is satisfied, so that we can prove the time complexity of the algorithm to be *O*(*nk*). Although we assume that the block diversity evaluation function we used here is monotonic, we can modify the algorithm slightly such that it can be applied to nonmonotonic blocks. In the case of nonmonotonic blocks, for each SNP *i*, we use *L*_*i*_ to denote the set of all *x* such that [*x*, *i*] is a feasible haplotype block. Let *L* = *nl* = ∑_*i*=1_^*n*^|*L*_*i*_|, where *l* is the average number of |*L*_*i*_| for each marker *i*. It can be shown that the modified algorithm uses *O*(*kn* *l*) time and *O*(*nl*) space.

### 3.3. A Linear Space Algorithm for Haplotype Block Partitioning with Limited Number of Tag SNPs

Using a similar concept as in [31], we find a cut point x* to divide *n* SNP sites into two parts, *n*_1_ and *n*_2_, and use t* tag SNPs for *n*_1_ and the other *t* − t*tag SNPs for *n*_2_ such that the total size of blocks covered by t* tags in *n*_1_ and *t* − t*tags in *n*_2_ is maximized. We obtain the following recurrence relation:

(9) $$\begin{array}{c} f\left(i,j,t\right)\;=\;f\left(i,x^{*},t^{*}\right)\;+\;f\left(x^{*}+1,j,t-t^{*}\right). \end{array}$$

**Figure 5:**
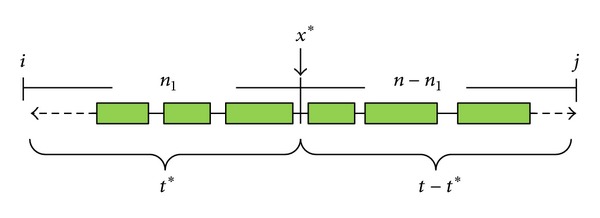
Illustration of the idea of recurrence *f*(*i*, *j*, *t*).

The idea behind the recurrence relation is illustrated in Figure 5. Note that in order to maximize the total size of blocks tagged by t* SNPs and *t* − t*SNPs, we are unable to assign half of *t* to t* directly, because in some cases the ⌊*t*/2⌋th and the (⌊*t*/2⌋ + 1)th SNP will be used to tag the same block that is a member of the longest segmentation. If we use the first to the ⌊*t*/2⌋th SNPs to tag the blocks in *n*_1_ and use the (⌊*t*/2⌋ + 1)th to the *t*th SNPs to tag the blocks in *n*_2_, we will not obtain the longest segmentation for *n* SNPs. In the general case there will be many pairs of t* and x* solutions that satisfy our requirement. For time efficiency, we want to make x* and t* approximate half of *n* and *t* as nearly as possible. Let *t*_0_ denote the maximum number of tag SNPs required among all feasible blocks. In order to find the appropriate value of x* and t*, we can examine t* in *t*_0_ continuous possible values, ⌊*t*/2⌋ − ⌊*t*_0_/2⌋ ≤ t* ≤ ⌊*t*/2⌋ + ⌊*t*_0_/2⌋ and examine x* in all SNPs loci for each selection of t*. Since *t*_0_ is small in the general case, we can find x* and t* quickly. After finding appropriate values of x* and t*, we can execute the steps recursively to partition the original problem to two subproblems repeatedly. Until *t* ≤ *t*_0_, we simply use the dynamic programming algorithm to solve each subproblem. The algorithm traces back to output the boundaries of each block. The algorithm is shown in Algorithm 3.

**Theorem 7 (longest-blocks-*t*-tags)**

Assume that the maximum number of tag SNPs required among all feasible blocks, *t*_0_, is a constant. Given a haplotype matrix *A*, a diversity upper limit *D*, and a number of tag SNPs t, find a segmentation S consisting of k feasible blocks such that (for all *i*) (*δ*(*Bi*) ≤ *D*) and ∑tag  (*Bi*) ≤ *t*, so that maximizing the total length of *S* can be done in *O*(*t* *nl*) time and using linear space after the preprocessing of *R*_*i*_, *L*_*i*_, and tag(*k*, *i*)*'s*, *k* ∈ *L*_*i*_, for each SNP *i*.

**Figure 6:**
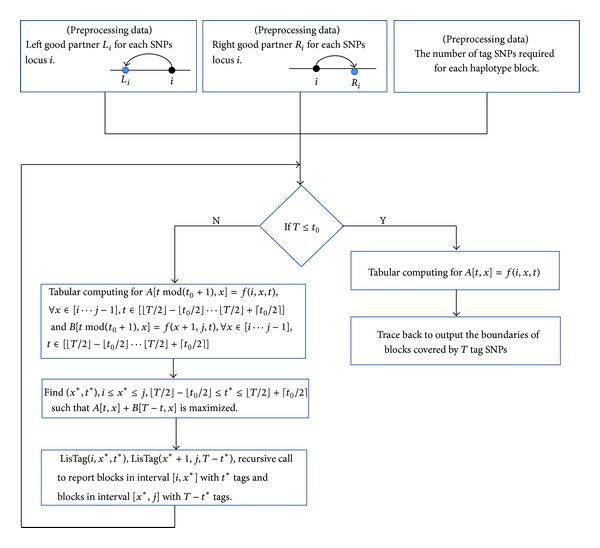
The flowchart of HBPTS.

In the algorithm, named HBPTS, we use five global data structures involving arrays *E*, *F*, *S*, *A*, and *B*. Arrays *E* and *F* are used to store the good partner points *L*_*i*_ and *R*_*i*_ for each SNP *i*, and array *S* is used to store the tag SNPs required for each feasible block. The data in arrays *E*, *F*, and *S* were calculated in preprocessing, and the size of each array is *L*, the number of all feasible blocks. In addition, we use a two-dimensional array *A* for computing the *f*(*i*, *x*, 0 ⋯ ⌊*T*/2⌋ + ⌊*t*_0_/2⌋) and *B* for computing the *f*(*x* + 1, *j*, 0 ⋯ ⌊*T*/2⌋ + ⌊*t*_0_/2⌋). Note that the computation of *f*(*i*, *j*, *t*) will compare the values of *f*(*i*, *k* − 1; *t* − tag(*k*, *j*)), *k* ∈ *L*_*j*_, and *f*(*i*, *j* − 1, *t*). Therefore, if *t*_0_ denotes the maximum tag(*k*, *j*), the maximum number of tag SNPs required among all feasible blocks, we need to store at most the values of *f*(…, …, (*t* − *t*_0_) ⋯ (*t* − 1)) and *f*(*i*, *j* − 1, *t*) while computing the value of *f*(*i*, *j*, *t*). In our experience, the *t*_0_ will be equal to 8 at most, as seen, for example, in the haplotype data of Patil et al. [7]. Thus the space of two dimensional arrays *A* and *B* is *t*_0_ × *n*, so the space complexity for the algorithm is *O*(*L* + *t*_0_ *n*). Since *t*_0_ is generally a constant and *L* > *n* in most practical cases, we can prove that the space used by the algorithm is *O*(*L* + *n*). The flowchart of HBPTS is shown in Figure 6.

**Proof**

We propose an *O*(*t* *nl*) time algorithm, HBPTS(*i*, *j*, *T*), shown in Algorithm 2. The time complexity of the algorithm is *O*(*nl* *t*) as shown in the following by induction. Let *T*(*n*, *t*) denote the time needed for HBPTS(1, *n*, *t*). Assume that *T*(*n*_0_, *t*_0_) ≤ *c*_2_ *n*_0_ *lt*_0_ for all *n*_0_ < *n*, *t*_0_ < *t*. According to the algorithm, we have

(10) $$\begin{array}{c} T\left(n,t\right)=c_{1}nlt+T\left(n_{1},t^{*}\right)+T\left(n-n_{1},t-t^{*}\right). \end{array}$$

By induction,

(11) T(n,t)≤c1nlt+T(n1,t∗)+T(n−n1,t−t∗)≤l(c2nt+c1nt+2c2n1t∗−c2n1t−c2nt∗)≤l[c2nt+(53c2n1t∗−c2n1t)  +(c1nt+13c2n1t∗−c2nt∗)]≤l[c2nt+c2n1(53t∗−t)  +(c1nt+13c2n1t∗−c2nt∗)]≤l{c2nt+c2n1[53(⌊t2⌋+⌈t02⌉)−t]  +(c1+13c2)nt−c2n(⌊t2⌋−⌈t02⌉)}≤l{c2nt+c2n1[53(t2+t02+1)−t]  +(c1+13c2)nt−c2n(t2−t02−1)}≤l[c2nt+c2n1(56t0+53−16t)  +c2n(t02+1−t10)]≤c2nlt.

Letting *t* ≥ 5*t*_0_ + 10, the above inequality will be satisfied, so that we can prove the time complexity of the algorithm to be *O*(*nl* *t*).

## 4. Experiments

We applied our dynamic programming algorithms, which find the longest segmentation covered by a specific number of tag SNPs, to the haplotype data for chromosome 21 provided by Patil et al. [7]. The data contain 20 haplotype samples and each contains 24,047 SNPs spanning 32.4 Mb of chromosome 21. The minor-allele frequency at each marker locus is at least 10%. Using the proposed algorithms with the same criteria as in [7] with ≥80% coverage of common haplotypes in blocks, 3,260 tag SNPs and 2,266 haplotype blocks are identified. In contrast, 4,563 tag SNPs and 4,135 blocks are identified in [7], and 3,582 tag SNPs and 2,575 blocks are identified in [10]. The proposed algorithms reduce the number of tag SNPs and blocks by 28.6% and 45.2% compared to [7]. We also demonstrate that the results shown in [10] are not optimal.

Table 1 shows a comparison of properties of haplotype blocks found by [7, 10] and our algorithms with 80% coverage of common haplotypes. The proposed algorithms discover 736 blocks containing more than 10 SNPs per block. Blocks with >10 SNPs account for 32.5% of all blocks. The average number of SNPs for all of the blocks is 10.6. The largest block contains 128 common SNPs, which is longer than the largest block (containing 114 SNPs) identified by [7] and the same as identified by [10]. Tables 2 and 3 show more experimental results. According to these results, we can partition 38.6% of the genome region into blocks that require no tag SNPs. This is because most of these blocks contain only a few common SNPs, and 80% of the unambiguous haplotypes have the same haplotype pattern (are compatible) in these blocks. We term these SNP loci as uninformative markers because they are the same among most (80%) of the population. These data also show that as the covered genome region increases, we need to add more and more tag SNPs to capture the haplotype information of the blocks, and the number of zero-tagged blocks becomes fewer. Although the average length of non-zero-tagged blocks becomes shorter as the covered chromosome region increases, the average length of all blocks becomes longer.

**Figure 7:**
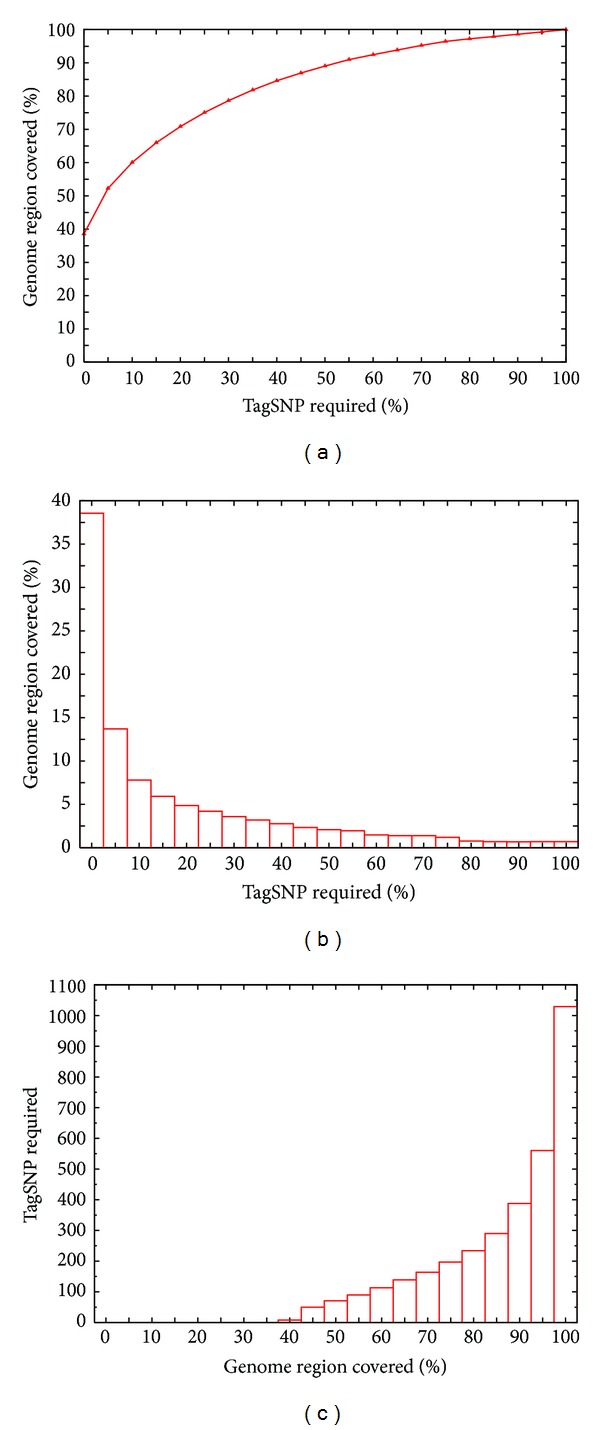
(a) The percentage of genome region covered by the percentage of tag SNPs, (b) the increase in percentage of covered genome region corresponding to a 5% increase in number of tag SNPs, and (c) the increase in number of tag SNPs needed to increase the covered genome region by 5%.

Figure 7(a) shows the percentage of tag SNPs identified by the proposed algorithms when blocks cover a specified percent of the genome region. According to our experimental results, when blocks cover 70% of the genome region, the proposed algorithm required only 19.1% (about 623) of the tag SNPs to capture most of the haplotype information. This also indicates that the proposed method discovers that only a few tag SNPs are needed to capture most of the genome-region information.  Figure 7(b) shows that the percentage of covered genome region increases while the tag SNPs identified by the proposed methods increase by 5%. Note that as the number of tag SNPs increases, the marginal percentage of genome region covered decreases. This indicates that, as the genome region coverage increases, fewer common SNPs are covered by each tag SNP on average.  Figure 7(c) shows the added tag SNPs needed to increase the genome-region coverage by 5%. We find that as genome-region coverage increases, many more tag SNPs are needed to capture the haplotype information. In particular, when the genome-region coverage increases from 95% to 100%, we need to use another extra 1,014 tag SNPs, about 31.1% of the total tag SNPs. It is interesting to note that the proposed method discovers that the marginal utility of tag SNPs decreases as genome-region coverage increases. From the results, our algorithms obtain better results than those by the other methods [7, 10] on the same haplotype sample. One of the main reasons is that their algorithms presume that the common-haplotypes evaluation function satisfies the monotonic property. However, when the haplotype sample has missing data, the diversity function does not satisfy the monotonic property. For example, Table 5 shows the analysis results of Zhang's and our algorithms on the same haplotype sample; this sample has just 69 SNPs, which is a small part of Patil's haplotype data [7]; the contig number is NT 001035. Using the sample criteria (80% of common haplotype), our methods partition the sample into 20 blocks and identify 18 tag SNPs, whereas Zhang's algorithm partitions the sample into 23 blocks and uses 22 tag SNPs. The results are similar in interval [21840, 21875], but in interval [21876, 21899], our methods discover 3 blocks and 3 tag SNPs, whereas Zhang's gives 6 blocks and 6 tag SNPs. In interval [21900, 21908], both Zhang's and our methods find 2 blocks, but our method needs only 3 tag SNPs rather than the 4 found by Zhang's method. These cases demonstrate that Zhang's algorithm fails to find the optimal solution owing to the nonmonotonic property of the common-haplotype evaluation function.

We also apply our algorithm on biological data set from chromosome 20 from HapMap data bulk (http://www.hapmap.org), respectively. The data set contains 120 individuals that include 71,539 SNPs from the Yoruba in Ibadan, Nigeria (abbreviated YRI). We select the first 30 individuals and the first 5,000 SNPs for the input sample of the algorithm. Using the diversity function (3) with ≥80% coverage of common haplotypes in blocks, the total number of blocks and tag SNPs identified by the algorithm is 293 and 1,047. The haplotype sample also being applied to Zhang's method with minimum number of tag SNPs implemented on HapBlock [23], using the same criteria, 344 blocks and 1,184 tag SNPs are obtained by Zhang's algorithm.

Table 4 shows a comparison of properties of haplotype blocks identified by Zhang's and our algorithms with 80% coverage of common haplotypes. Our algorithm discovers 168 blocks containing more than 10 SNPs per block. Blocks with >10 SNPs account for 57.3% of all blocks. The average number of SNPs for all of the blocks is 17.1. The largest block contains 92 common SNPs, which is longer than the largest block (containing 79 SNPs) identified by Zhang's algorithm. Note that the haplotype sample has no missing data, and using the data set, the diversity function (3) will satisfy the monotonic property.

## 5. Conclusion

In this paper, we examine several haplotype diversity evaluation functions. By use of appropriate diversity functions, the block selection problem can be viewed as finding a segmentation of a given haplotype matrix such that the diversities of chosen blocks satisfy a given value constraint. Tag SNPs can capture most of the haplotype diversity in the blocks and thereby can potentially capture most of the information for association between a trait and the SNP marker loci. Instead of genotyping all of the SNPs on the chromosome, one may wish to use only the genotype information of tag SNPs. We can infer the haplotype features of most populations by genotyping only a few SNPs. Thus, identifying tag SNPs can dramatically reduce the time and effort needed for genotyping, without loss of much haplotype information.

We present two dynamic programming algorithms for haplotype-block partitioning such that total block length is maximized and the total tag SNPs required are minimized. We also show in Theorem 6 that finding the longest *k*-block segmentation with diversity constraints can be done in *O*(*nk*) time and *O*(*n*) space. In Theorem 7, we show that finding a maximum segmentation with limited tag SNPs number can be done in *O*(*nl* *t*) time; furthermore, we reduce the space complexity into *O*(*L* + *n*). We point out that these efficiency results of our algorithms can be applied in many different definitions of diversity functions, provided that we can precompute the boundaries of all feasible blocks and tag SNPs required for these blocks.

We also show that the experimental results discovered by our methods are superior to those by Zhang's algorithm. We demonstrate that owing to the nonmonotonic property of the common-haplotype evaluation function, Zhang's algorithm will not find an optimal solution when the haplotype samples have missing data.

## Figures and Tables

**Algorithm 1:**
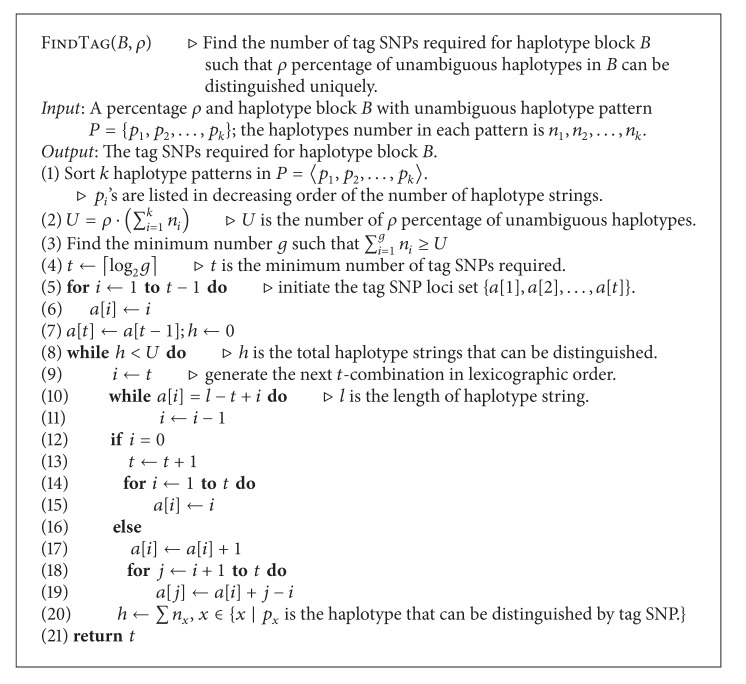
The exhaustive searching algorithm for tag SNP selection.

**Algorithm 2:**
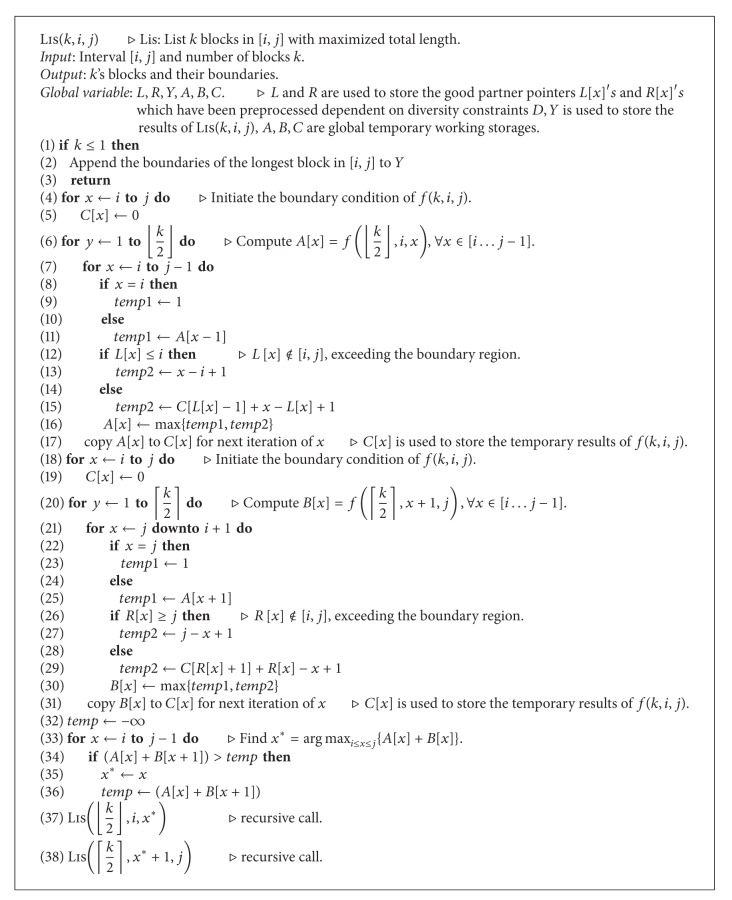
The *O*(*nk*) time and linear space algorithm for haplotype blocking.

**Algorithm 3:**
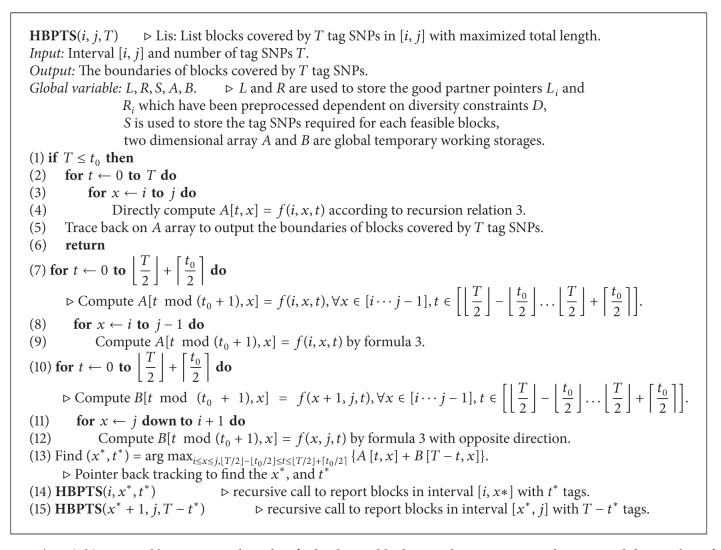
The *O*(*nl* *t*) time and linear-space algorithm for haplotype blocking with constraints on diversity and the number of tag SNPs.

**Table 1:** Comparison of properties of haplotype blocks defined by Zhang, Patil, and us with 80% of common haplotype coverage, using the chromosome 21 haplotype sample proposed by Patil.

Method	Common SNPs per block	No. of blocks	No. of blocks requiring ≥1 tagSNPs	Average no. of common haplotype per block	Block frequency (%)	SNP frequency (%)
HBPTS	>10	736	733	4.32	32.5	77.8
	3–10	751	686	3.16	33.1	18.0
	<3	779	216	2.12	34.4	4.2
	**Total**	**2,266**	**1,635**	**3.18**	**100.0**	**100.00**

Zhang's	>10	742	738	4.23	28.8	75.5
	3–10	909	842	3.03	35.3	19.5
	<3	924	274	2.12	35.9	5.0
	**Total**	**2,575**	**1,854**	**3.05**	**100.0**	**100.0**

Patil's	>10	589	589	3.75	14.2	56.8
	3–10	1,408	1,396	2.92	34.1	30.7
	<3	2,138	1,776	2.30	51.7	12.4
	**Total**	**4,135**	**3,761**	**2.72**	**100.0**	**100.0**

**Table 2:** Analysis results based on numbers of tag SNPs required (using the chromosome 21 haplotype sample proposed by Patil).

Tag SNPs used	Genome region covered (%)	Extra genome region increased (%)	0-tagged blocks	Blocks with tags number >0	Avg. length of non-0-tagged blocks
0% (0)	38.55	38.55	6136	0	1.51 (0-tagged blocks)
10% (326)	59.99	21.44	4991	192	35.52
20% (652)	70.85	10.86	4145	367	29.37
30% (978)	78.62	7.77	3387	516	26.79
40% (1304)	84.61	5.99	2897	712	22.38
50% (1630)	89.02	4.41	2250	844	21.29
60% (1956)	92.59	3.57	1814	1002	19.41
70% (2282)	95.30	2.71	1478	1159	17.79
80% (2608)	97.29	1.99	1014	1289	16.90
90% (2934)	98.64	1.35	719	1421	15.89
100% (3260)	100.00	1.36	631	1635	14.10

**Table 3:** Analysis results based on percentage of genome region covered (using the chromosome 21 haplotype sample proposed by Patil).

Genome region covered (%)	Tag SNPs required	Extra tag SNPs required	0-tagged blocks number	Blocks with tags number >0	Avg. length of non-0-tagged blocks
38.55	0	0	6136	0	1.51 (0-tagged blocks)
40	8	8	6111	6	67.17
50	127	119	5630	80	43.75
60	327	200	4991	193	35.39
70	623	296	4213	347	30.22
80	1045	422	3307	567	25.14
90	1709	664	2208	888	20.58
100	3260	1551	631	1635	14.10

**Table 4:** Comparison of properties of haplotype blocks defined by Zhang and us with 80% of common haplotype coverage, using the chromosome 20 haplotype sample from HapMap.

Method	Common SNPs per block	No. of blocks	No. of blocks requiring ≥1 tagSNPs	Block frequency (%)	SNP frequency (%)
HBPTS	>10	168	168	57.3	84.8
	3–10	103	103	35.2	14.5
	<3	22	21	7.5	0.7
	**Total**	**293**	**292**	**100.0**	**100.00**

Zhang's	>10	170	170	49.4	80.2
	3–10	141	141	41.0	18.9
	<3	33	33	9.6	0.9
	**Total**	**344**	**344**	**100.0**	**100.0**

**Table 5:** The experimental results of Zhang's and our algorithms on a small part of Patil's data.

Id	Zhang's result		Our result	
	(Start, end)	Tag used	(Start, end)	Tag used
1	(21840, 21840)	0	(21840, 21840)	0
2	(21841, 21842)	1	(21841, 21843)	1
3	(21843, 21844)	0	(21844, 21844)	0
4	(21845, 21845)	0	(21845, 21845)	0
5	(21846, 21846)	0	(21846, 21846)	0
6	(21847, 21853)	3	(21847, 21853)	3
7	(21854, 21855)	1	(21854, 21855)	1
8	(21856, 21856)	0	(21856, 21856)	0
9	(21857, 21860)	2	(21857, 21860)	2
10	(21861, 21863)	2	(21861, 21863)	2
11	(21864, 21865)	1	(21864, 21865)	1
12	(21866, 21869)	1	(21866, 21869)	1
13	(21870, 21870)	0	(21870, 21871)	1
14	(21871, 21874)	1	(21872, 21874)	0
15	(21875, 21875)	0	(21875, 21875)	0
16	(21876, 21889)	2	(21876, 21876)	0
17	(21890, 21891)	0	(21877, 21882)	1
18	(21892, 21894)	1	(21883, 21899)	2
19	(21895, 21895)	1	(21900, 21907)	2
20	(21896, 21897)	0	(21908, 21908)	1
21	(21898, 21899)	2		
22	(21900, 21902)	2		
23	(21903, 21908)	2		

Total	23	22	20	18
